# Correlation of cumulus gene expression of *GJA1*, *PRSS35*, *PTX3*, and *SERPINE2* with oocyte maturation, fertilization, and embryo development

**DOI:** 10.1186/s12958-015-0091-3

**Published:** 2015-08-16

**Authors:** Sheng-Hsiang Li, Ming-Huei Lin, Yuh-Ming Hwu, Chung-Hao Lu, Ling-Yu Yeh, Ying-Jie Chen, Robert Kuo-Kuang Lee

**Affiliations:** Department of Medical Research, Mackay Memorial Hospital, Tamsui District, New Taipei City, 251 Taiwan; Mackay Junior College of Medicine, Nursing, and Management, Beitou District, Taipei City, 112 Taiwan; Graduate Institute of Biochemical and Biomedical Engineering, National Taipei University of Technology, Taipei City, 106 Taiwan; Department of Obstetrics and Gynecology, Mackay Memorial Hospital, Taipei City, 104 Taiwan; Mackay Medical College, Sanzhi District, New Taipei City, 252 Taiwan; Department of Obstetrics and Gynecology, Taipei Medical University, Taipei City, 110 Taiwan

**Keywords:** Gene expression, Oocyte, Cumulus cells, Embryo development

## Abstract

**Background:**

*GJA1* and *PTX3* were proposed as gene markers for oocyte and embryo developmental competence, while *SERPINE2* was reported to be associated with pregnancy outcome. *PRSS35*, which is exclusively expressed in the ovary, may be correlated with oocyte competence. This study was conducted to evaluate the correlation of cumulus *GJA1*, *PRSS35*, *PTX3*, and *SERPINE2* gene expression levels with oocyte maturation, fertilization, and early embryo development.

**Methods:**

In total, 308 cumulus cell samples separated from individual cumulus–oocyte complex were obtained from 40 patients undergoing the intracytoplasmic sperm injection treatment procedure. Gene expression levels (mRNA levels) in cumulus cells were assessed using quantitative real-time polymerase chain reaction.

**Results:**

Gene expression levels of *GJA1* and *SERPINE2* in cumulus cells surrounding mature oocytes were significantly lower than those in cumulus cells enclosing immature oocytes. *PRSS35* mRNA levels in cumulus cells of fertilized oocytes were significantly higher than those in cumulus cells of unfertilized oocytes. *GJA1* and *SERPINE2* seemed to express higher mRNA levels, while *PRSS35* showed lower expression in cumulus cells of oocytes that developed into embryos with good morphology; however, the expression levels of all three genes and *PTX3* showed no significant differences between embryos with good or poor morphology.

**Conclusions:**

*GJA1* and *SERPINE2* represent potential gene markers associated with oocyte maturation. *PRSS35* may be correlated with oocyte fertilization potential. However, *GJA1*, *PRSS35*, *PTX3*, and *SERPINE2* may not be considered as marker genes for predicting embryo morphology.

## Background

Oocytes secrete paracrine factors such as growth differentiation factor 9 and bone morphogenetic protein 15, which affect the gene expression of cumulus cells, thereby leading to cumulus expansion [1, 2]. Cumulus expansion involves hyaluronan accumulation in the intercellular space of cumulus cells [3], and the structural integrity of the cumulus cell extracellular matrix (ECM) is essential for oocyte maturation. Several cumulus proteins linked to ECM hyaluronan, including pentraxin-3 (PTX3) [4, 5], are required for cumulus integrity, thereby ensuring cumulus expansion and oocyte maturation. In addition, nutrients and metabolites are transported through gap junctions between the oocyte and cumulus cells [6]. Taken together, bidirectional intercellular communication between oocytes and their surrounding cumulus cells is essential for the development of an egg that is competent to undergo fertilization and embryo development [3, 7, 8].

Cumulus cells gene expression may reflect oocyte maturation and competence. Numerous studies have been conducted to profile cumulus gene expression, identify gene markers, and predict oocyte or embryo competence [9–11]. The expression of potential marker genes in cumulus cells has been suggested to be associated with oocyte competence [10–14], embryo quality [9, 15–17], pregnancy outcome [9, 18, 19], and live birth [20].

GJA1, also known as connexin 43, is the major gap junction protein that is expressed in cumulus granulosa cells, participating in connexons with other cumulus granulosa cells or the oocyte. GJA4, corresponding to connexin 37, appears to be the only connexin synthesized by the oocyte; it forms a heterologous gap junction with granulosa cells [6]. Loss-of-function of *GJA4* interferes with the development of antral follicles [21, 22] and can be rescued by replacement with *GJA1* [23]. *GJA1* has been identified as a gene marker for oocyte fertilization potential and embryo quality [16, 17].

*PTX3* gene expression in cumulus cells was also associated with oocyte/embryo competence and was a potentially reliable predictor of embryo developmental competence [14].

Proteases play an important role in the proteolysis process and are essential for tissue remodeling and functions of the ovary in which extensive tissue remodeling during folliculogenesis and ovulation take place. *SERPINE2* mRNA levels in granulosa cells have been suggested to be a potential pregnancy biomarker [18]. Higher *SERPINE2* expression levels were detected in cumulus cells of human immature oocytes than in those of mature oocytes [24, 25]. *PRSS35* belongs to the trypsin class of serine proteases. This protease is exclusively expressed in the mouse ovary, suggesting that *Prss35* is involved in ovarian functions [26].

In the present study, we focused on the four cumulus-expressed genes *GJA1*, *PRSS35*, *PTX3*, and *SERPINE2* and evaluated whether their expression levels correlate with oocyte maturation, fertilization, and embryo development in humans.

## Methods

### Patients and cumulus cell collection

Patients (*n* = 40) undergoing intracytoplasmic sperm injection (ICSI) treatments at the Center of Reproductive Medicine, Mackay Memorial Hospital, Taiwan received controlled ovarian hyperstimulation using the gonadotropin-releasing hormone antagonist protocol as described previously [27]. All patients were younger than 38 years. The number of oocytes retrieved per patient was larger than 5. Sperm samples collected from patients with the following criteria were excluded from the study: sperm counts ≤ 100,000/ml, sperm motility ≤ 10 %, and sperm with normal morphology < 1 %. Patients with oocyte fertilization rates < 30 % in the previous ICSI treatments were also excluded. This study was approved by the Mackay Memorial Hospital Institutional Review Board (reference number 09MMHIS024), and written consent for the use of human cumulus cells was obtained.

**Fig. 1:**
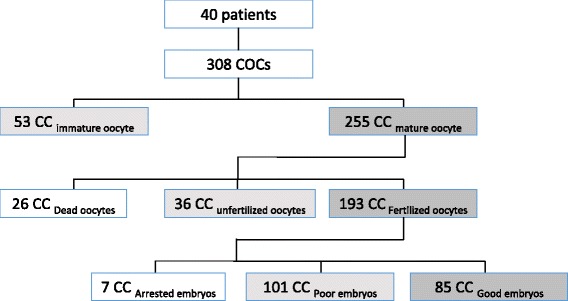
Classification of cumulus cell samples used in this study. In total, 308 cumulus–oocyte complex (COC) samples were collected from 40 patients who underwent the ICSI procedure. Fertilized oocytes were cultured to day 3, and the early embryo morphology was assessed as described in “Materials and Methods”. CC indicates the cumulus cells

The cumulus–oocyte complexes (COCs) in antral follicles with a diameter larger than 14 mm were collected using transvaginal ultrasound and a 16-gauge needle and were exposed to 80 IU hyaluronidase in Quinn’s Advantage Fertilization medium (Sage BioPharma, Bedminster, NJ) for 20 s at 37 °C to dissolve hyaluronan. The cumulus cells were individually collected from COCs under an Olympus SZX7 stereomicroscope (Tokyo, Japan). In total, 308 COCs were collected. The classification of cumulus cell samples is shown in Fig. 1. Samples were mixed with 20 μl of extraction buffer from the Arcturus PicoPure RNA Isolation Kit (Applied Biosystems, Foster City, CA) for total RNA isolation and stored at −80 °C until use.

### Assessment of oocyte maturity, fertilization, and embryo quality

Oocytes with the first polar body extrusion were defined as mature oocytes; otherwise, they were defined as immature oocytes. Fertilization was assessed by the presence of two pronuclei and two polar bodies at 16–20 h (day 1) after ICSI. Embryos were morphologically graded on the basis of the degree of cellular fragmentation and the regularity of blastomere size. The day 3 embryo quality scoring system, modified from previous reports [28, 29], was used to grade the embryo as follows: grade 1, even-sized blastomeres with no cytoplasmic fragmentation; grade 2, even-sized blastomeres with minor cytoplasmic fragmentation ≤ 20 % of the embryo surface; grade 3, uneven-sized blastomeres with variable fragmentation; grade 4, even—or uneven-sized blastomeres with moderate to significant cytoplasmic fragmentation > 20 % of the embryo surface; and grade 5, few blastomeres of any size and severe fragmentation ≥ 50 % of the embryo surface. Grade 1–2 embryos were considered to be good quality embryos, while grade 3–5 embryos were considered to be poor-quality embryos.

### Total RNA extraction, cDNA synthesis, and quantitative real-time polymerase chain reaction (qPCR)

Total RNA of cumulus cells collected from a single COC was extracted using the Arcturus PicoPure RNA Isolation Kit and directly reverse transcribed into a 20 μl first-strand cDNA pool using the High Capacity cDNA Archive Kit (Applied Biosystems) according to the manufacturer’s instructions. qPCR was performed in a total volume of 20 μl, containing equally distributed cDNA, 250 nM each of the forward and reverse primers (Table 1), and 10 μl of 2× SYBR Green Master Mix (Applied Biosystems). All reactions were performed in triplicate and run on the ABI/PRISM 7500 Fast Sequence Detection System (Applied Biosystems) under the following conditions: 95 °C for 20 s, followed by 40 cycles at 95 °C for 1 s and 60 °C for 20 s. The PCR amplification efficiency of each tested gene was pretested to ensure that it was equivalent to that of the housekeeping gene examined in a cDNA dilution series. The threshold cycle (Ct) was defined as the fractional cycle number at which the reporter fluorescence that is the number of amplified copies reached a fixed threshold. Melting curve analysis was performed in order to verify that only a single product had formed in the reaction. DNA sequencing was performed to confirm the identity of PCR products. Relative quantification of mRNA expression was calculated using the 2^−ΔΔCt^ method [30]. The housekeeping gene *RPL19*, encoding ribosomal protein L19, was used as the internal loading control to normalize the relative gene expression levels as it was validated as a suitable reference gene [31].

**Table 1:** Summary of real-time PCR primers

Gene name	GenBank accession no.	Primer sequence (5′ to 3′)	Product size (bp)
*GJA1*	NM_000165	F^a^: AGCAGTCTGCCTTTCGTTGTAAC	94
		R^b^: ACCCAGAAGCGCACATGAG	
*PRSS35*	NM_001170423.1	F: ATTTGCCTCTGGATTCACGG	110
		R: CGGTAAGCAGAGCTGGTTTTCT	
*PTX3*	NM_002852.3	F: TCCATCCCACTGAGGACCC	102
		R: TGCGCTCTCTCATCTGCG	
*SERPINE2*	NM_001136528	F: TCTCATTGCAAGATCATCGCC	97
		R: CCCCATGAATAACACAGCACC	
*RPL19*	NM_000981	F: TCGATCGCCACATGTATCACAG	93
		R: TCAGCTTGTGGATGTGTTCCA	

^a^ F, forward primer; ^b^ R, reverse primer

### Statistical analysis

The nonparametric Mann–Whitney test was used for data analysis to compare the variables between two independent groups without normal distribution. The median value of each dataset is shown. *P* values of <0.05 were considered significantly different. GraphPad Prism 5 (San Diego, CA) was used for all analyses.

## Results

We measured the gene expression levels of *GJA1*, *PRSS35*, *PTX3*, and *SERPINE2* in cumulus cells collected from a single COC. We also recorded oocyte maturity status, fertilization outcome of oocytes after ICSI, and morphology of day 3 embryos developed from fertilized oocytes. Statistical analyses were performed to evaluate the correlations between gene expression levels and oocyte maturation, fertilization, and embryo development.

### *GJA1* and *SERPINE2* mRNA levels in cumulus cells correlated with oocyte maturation

**Fig. 2:**
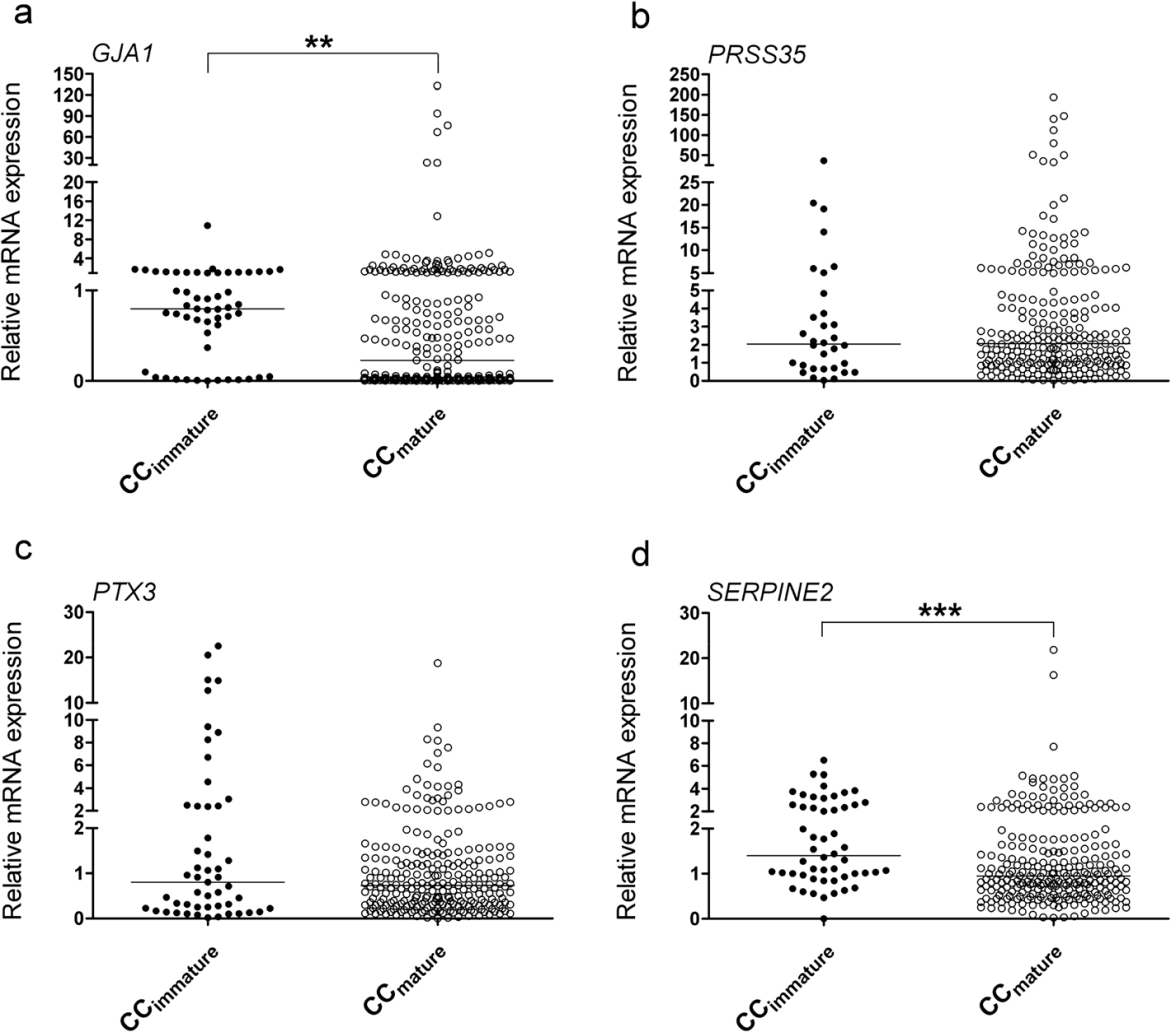
qRT-PCR revealed relative mRNA levels of *GJA1* (**a**), *PRSS35* (**b**), *PTX3* (**c**), and *SERPINE2* (**d**) in cumulus cells surrounding immature or mature oocytes. **P < 0.01; ***P < 0.001

Cumulus cells surrounding mature oocytes expressed significantly lower mRNA levels of *GJA1* and *SERPINE2* than those from immature oocytes (*P* = 0.0034 and *P* = 0.0002, respectively). On the other hand, *PRSS35* and *PTX3* mRNA levels did not show significant differences (Fig. 2).

### Cumulus-expressed *PRSS35* mRNA levels correlated with fertilization

**Fig. 3:**
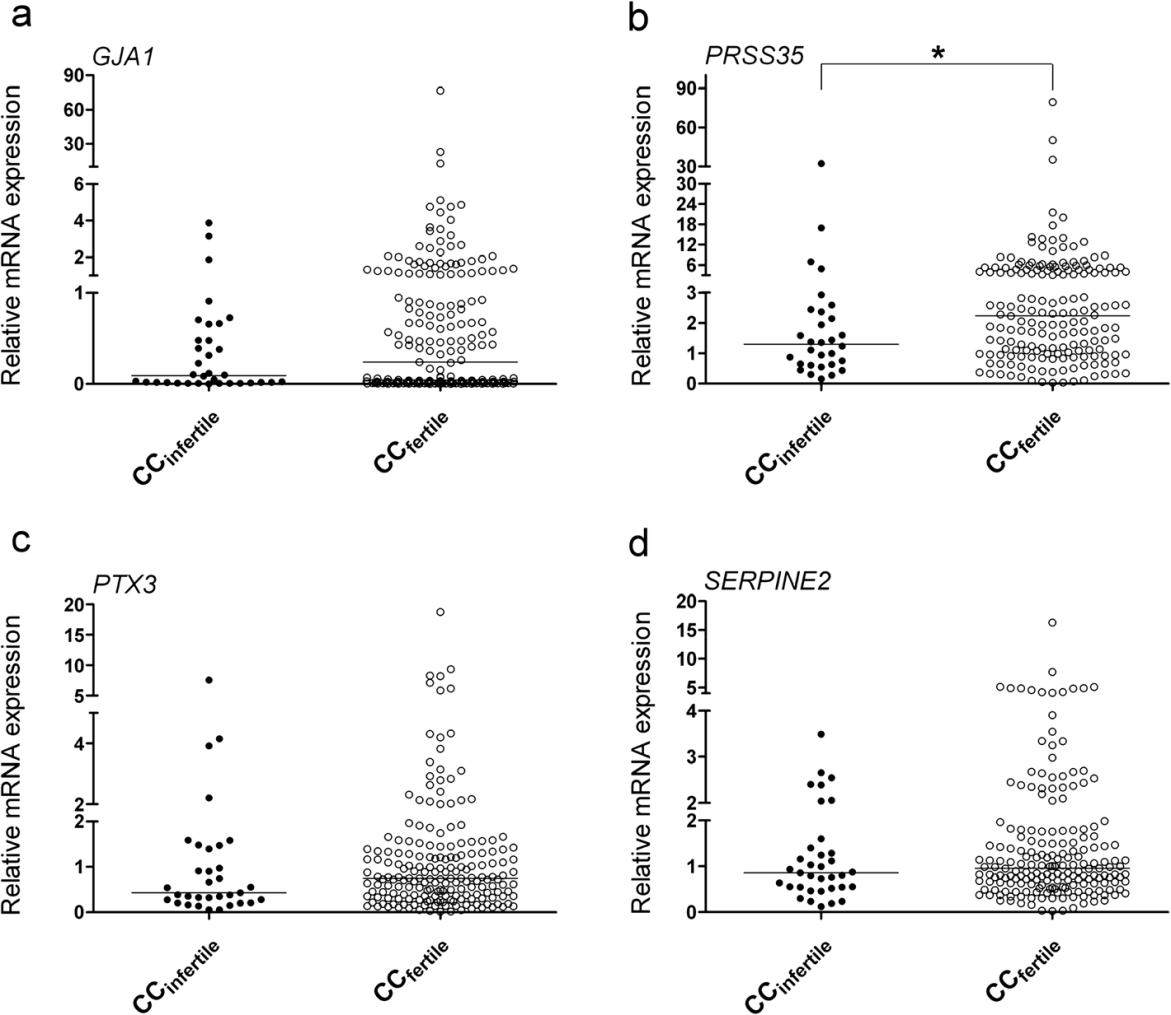
Relative mRNA levels of *GJA1* (**a**), *PRSS35* (**b**), *PTX3* (**c**), and *SERPINE2* (**d**) in cumulus cells of fertilized and unfertilized oocytes, as revealed by qRT-PCR. *P < 0.05

Cumulus cells collected from fertilized mature oocytes seemed to express higher mRNA levels of *GJA1*, *PRSS35*, *PTX3*, and *SERPINE2* than those collected from unfertilized oocytes; however, only *PRSS35* showed significant expression levels (*P* = 0.0188, Fig. 3).

### *GJA1*, *PRSS35*, *PTX3*, and *SERPINE2* expression levels were not significantly different between embryos with good and poor morphology

**Fig. 4:**
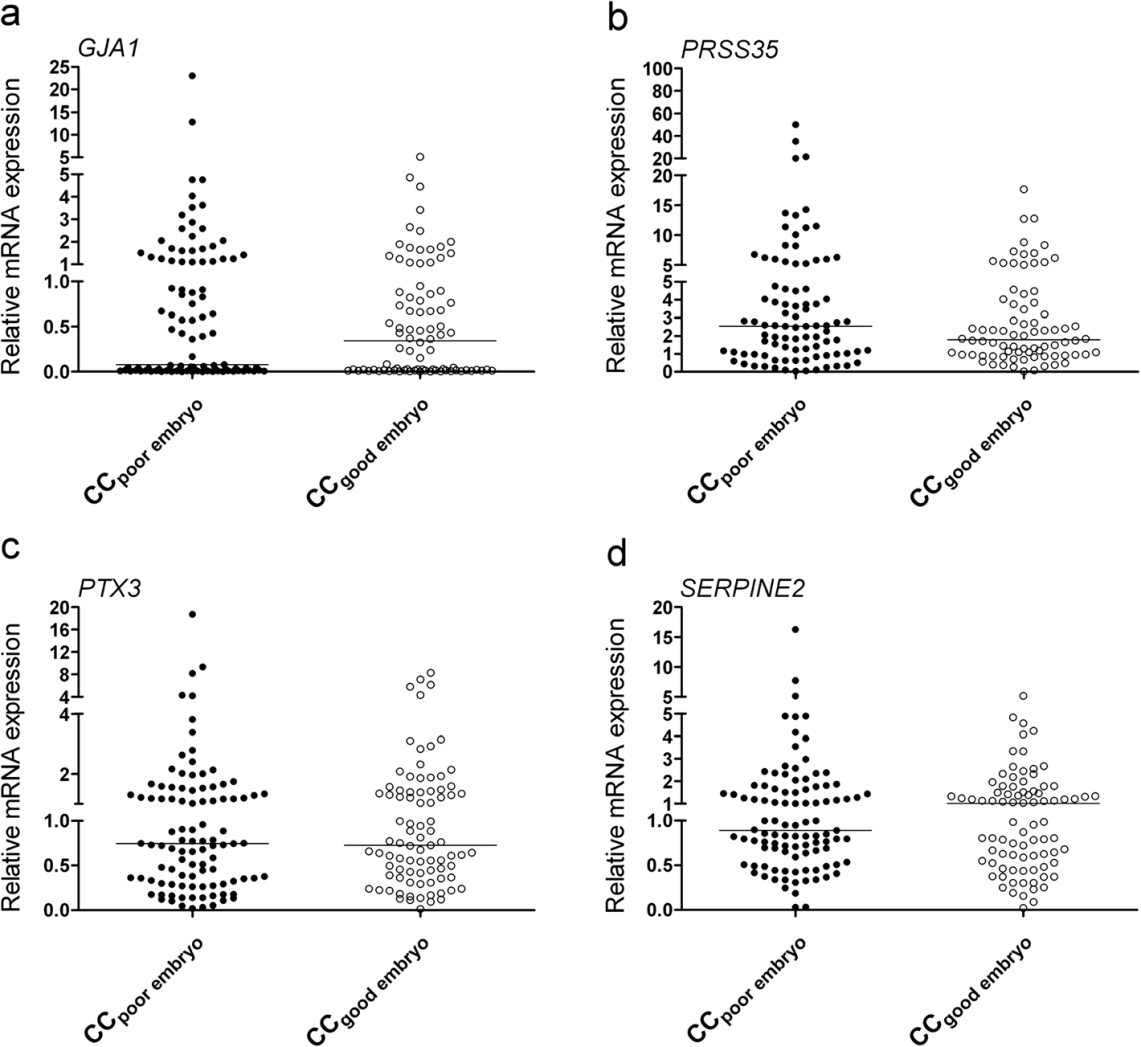
qRT-PCR revealed the relative mRNA levels of *GJA1* (**a**), *PRSS35* (**b**), *PTX3* (**c**), and *SERPINE2* (**d**) in cumulus cells collected from oocytes developing into embryos with good or poor morphology

It seemed that *GJA1* and *SERPINE2* were expressed at higher mRNA levels and *PRSS35* was expressed at lower mRNA levels in cumulus cells encircling mature oocytes developing into high-quality embryos (grades 1 and 2) than in cumulus cells encircling mature oocytes developing into poor-quality embryos (grades 3–5); however, the difference was not statistically significant (Fig. 4a, b, and d). There was no difference in *PTX3* expression (Fig. 4c).

## Discussion

GJA1-mediated gap junctional communication regulates oocyte meiosis resumption, and lower levels of GJA1 in cumulus cells are beneficial for oocyte maturation [32]. Our data showed that there was significantly lower expression of *GJA1* in cumulus cells of mature oocytes than in those of immature oocytes. Feuerstein et al. reported a similar result [12]; however, no difference was found in a study with a relatively smaller sample size [16].

*GJA1* mRNA levels in cumulus cells showed no significant differences between fertilized and unfertilized oocytes (Fig. 3), in line with the results reported by Hasegawa et al. [16]. *GJA1* expression in cumulus cells surrounding the mature oocytes did not show any difference in developing embryos with good or poor morphology (Fig. 4), as reported previously [12]; however, significantly lower expression was also reported for embryos with good morphology [16]. Intriguingly, while *GJA1* expression in cumulus cells enclosing oocytes achieving blastocyst development showed significantly lower levels than that in cumulus cells enclosing oocytes unable to develop to blastocyst stage [12], its expression in cumulus cells was also reported to be significantly higher in pregnant patients [17].

*SERPINE2* mRNA levels in cumulus cells are associated with oocyte maturation, with a significantly lower level in mature oocytes [24, 25]. This correlation was verified in our study (Fig. 2) using a larger sample cohort. In a mouse animal model, higher levels of SERPINE2 were demonstrated to impair cumulus expansion and oocyte maturation [25]. Although SERPINE2 showed a trend toward a higher expression in cumulus cells of oocytes developing into good embryos than in cumulus cells of oocytes developing into poor embryos (Fig. 4), the difference was not significant between the two groups. Nevertheless, *SERPINE2* mRNA levels were significantly higher in granulosa cells collected from follicles that resulted in pregnancy [18].

Our data revealed that *PRSS35* mRNA levels are associated with oocyte fertilization potential (Fig. 2). This is the first report demonstrating a relationship between human *PRSS35* expression and oocyte competence. *Prss35*-null mice have no defects in female fertility, suggesting that the gene is dispensable for murine fertility and embryo development [33]. However, this remains unclear in case of humans. Diao et al. did not detect any compensatory upregulation of other proteases reported in the uterus; however, the expression of other protease-related genes cannot be ruled out [33]. Alternatively, other compensatory effects may exist in *Prss35* knockout mice as PRSS35 may not possess any serine protease activity because the amino acids in its protease catalytic active site are changed. A study employing knockout mice lacking specific proteases that cause some unexpected results in the ovulatory process because of redundancy and overlapping functions has also been conducted previously [34].

While the expression of *PTX3* has been reported to be significantly lower in cumulus cells from immature oocytes than in those from mature oocytes [15], our data showed no significant difference (Fig. 2c). Pooled cumulus cells from fertilized oocytes were found to have 3- to 12-fold increases in *PTX3* levels than those from unfertilized oocytes [14]; however, the cumulus cells collected from individual COC in our study showed no significant difference. Congruent with previous reports [10, 20], we found that cumulus *PTX3* mRNA levels were not associated with the embryo morphological grade.

Selecting embryos with good morphology for transplanting has proved to be correlated with a good pregnancy outcome [35]. Our results showed that expression levels of *GJA1*, *PRSS35*, *PTX3*, and *SERPINE2* did not differ significantly between cumulus cells of embryos with good and poor morphology, indicating that these genes may not be used as marker genes for predicting early embryo development.

Comparison of cumulus gene expression of pregnant and nonpregnant embryo, which is the transfer of a single embryo, would be a good strategy for revealing good marker genes. Using the single embryo transfer (SET) strategy, several studies have proposed marker genes for pregnancy outcome [20, 36–38].

This study has some limitations: the controlled ovarian hyperstimulation procedure produces more mature oocytes than immature oocytes, and the ICSI treatment results in few unfertilized oocytes; thus, the sample size of immature oocyte or unfertilized oocyte is always small in this type of study, as seen previously [10, 12, 15, 16]. In addition, dead and arrested embryos are also found. Although cumulus cells that developed into good or poor embryos showed significantly higher *GJA1* mRNA levels than those that developed into arrested embryos (data not shown), the sample size was relatively less because of the unavailability of arrested embryos. Furthermore, this kind of study often produces contradictory results (e.g., *GJA1* and *PTX3* cases mentioned above). The cumulus expression of *EFNB2*, *RGS2*, and *VCAN*, which are proposed as biomarkers of pregnancy [19, 20, 36–38], was also inconsistent as revealed in a recently published report [39].

The selection of embryos with higher developmental potential has been one of the major challenges in assisted reproductive technology (ART). Because cumulus cells are discarded in ART, the cumulus samples have the advantage of being noninvasively collected. Although cumulus gene expression may represent a promising method compared with the currently used morphology-based method, more investigations are warranted. Thus, a prospective larger cohort study or the use of SET cumulus samples remains necessary to clarify the effectiveness.

In summary, *GJA1* and *SERPINE2* represent gene markers potentially associated with oocyte maturation, and *PRSS35* may be correlated with oocyte fertilization ability. *GJA1*, *PRSS35*, *PTX3*, and *SERPINE2* may not be considered as markers for predicting early embryo development.
